# Orchestration of B and T cell responses in health and disease by common gamma chain family cytokines with a focus on IL-21

**DOI:** 10.1186/ar3413

**Published:** 2011-09-16

**Authors:** Manfred Kopf, Luigi Tortola, Iwana Schmitz, Anja Fröhlich, Ivo Sonderegger, Helga Pawelski, Christoph Schneider

**Affiliations:** 1 Institute of Integrative Biology, Molecular Biomedicine, ETH Zürich, Switzerland

## 

Members of a subfamily of the type 1 four-helix-bundle cytokines with receptors sharing the common gamma (c_γ_) chain including IL-2, IL-4, IL-7, IL-9, IL-15, and IL-21 have distinct activities on the differentiation of effector, memory, and regulatory T cells [1,2]. Furthermore, IL-2, IL-4, and IL-21 serve distinct roles in control of B cell development and differentiation to antibody producing cells. We and others recently reported that both IL-2 and IL-21 are essential for maintenance of CD8 T cells and control of chronic viral infection, while both cytokines are dispensable for expansion and contraction of CD8 T cells during acute and resolved viral infection [3-7].

While IL-21 has been implicated in cross-regulation of Th17 cells and inducible regulatory T cells (Treg) *in vitro*, development of Th17 and Treg cells and consequently organ-related autoimmune disease remain unaffected in IL-21R-deficient mice *in vivo *[8,9]. In contrast, we now found that IL-21 can potently inhibit proliferation and function of inducible and natural Treg cells in models of T cell transfer colitis, viral infection, and asthma. Increased numbers of Tregs in IL-21R-deficient mice offer an explanation for suppression of Th2-mediated asthma and susceptibility to chronic viral infection described in the knockout mice [5,10].

Furthermore, the importance of IL-21 for B cell and antibody responses has been well established. Recently, it has been suggested that IL-21 is crucial for development of T follicular helper cells (Tfh) and defective B cell responses in IL-21R-deficient mice are due to the absence of Tfh cells. However, we found that germinal center development and antibody responses were severely impaired in mice that lack IL-21R specifically on B cells suggesting that IL-21 regulates germinal center responses in a B cell intrinsic manner [11]. In addition, we have shown that requirement of IL-21 for a B cell response is overcome by immunization with particulate antigens containing TLR7/8 ligands (such as viral RNS). These data demonstrate that innate pathogen patterns (PAMPs) and Th cell derived signals co-operate in the induction of optimal IgG responses. Interestingly, in contrast to follicular B cell responses, IL-21 has been shown to negatively regulate marginal zone (MZ) B-cell survival and antibody production to Streptococous pneumonia [12].
